# Preliminary results for the phase 1 trial of a folate receptor alpha adjuvant cancer vaccine in ovarian and endometrial cancer patients

**DOI:** 10.1186/2051-1426-1-S1-P200

**Published:** 2013-11-07

**Authors:** John S Berry, Erika J Schneble, Alfred F Trappey, Timothy J Vreeland, Guy T Clifton, Diane F Hale, Alan K Sears, Sathibalan Ponniah, Elizabeth A Mittendorf, George E Peoples

**Affiliations:** 1 General Surgery, San Antonio Military Medical Center, Fort Sam Houston, TX, USA; 2 General Surgery, Blanchfield Army Commmunity Hospital, Fort Campbell, KY, USA; 3 Cancer Vaccine Development Lab, USA Cancer Institute, Bethesda, MD, USA; 4 University of Texas, MD Anderson Cancer Center, Houston, TX, USA

## Background

Folate Receptor Alpha (FRa) is an immunogenic protein that is over-expressed in breast, endometrial and ovarian cancer (OC). In fact, FRa expression in malignant cells is 20-fold higher compared to normal cells. We have begun a phase 1 clinical trial with E39, an HLA-A2 restricted, FRa peptide vaccine. The vaccine is administered in the adjuvant setting to prevent recurrences in high-risk, endometrial and OC patients (pts) rendered clinically disease-free with standard-of-care therapy. Here, we summarize toxicity and in vivo immunologic responses after enrollment of three dose cohorts.

## Methods

The trial is being performed as a 3x3, dose-escalation, safety trial enrolling endometrial and OC pts. HLA-A2+ pts are enrolled into the vaccine group (VG) while HLA-A2- pts are being followed prospectively as an untreated control group (CG). Six monthly intradermal inoculations (R1-R6) of either 100mcg, 500mcg, or 1000mcg of E39 + 250 mcg GMCSF immunoadjuvant are administered during the primary vaccine series (PVS). Immunologic responses are assessed by both local reaction (LR) after each inoculation and delayed hypersensitivity (DTH) reaction measured pre-vaccination (R0) and after the PVS (R6). Recurrences are determined clinically. Data are means compared with a paired, t-test.

## Results

25 pts have enrolled; 13 in the VG and 12 in the CG. There are no significant differences in age, grade, stage, or nodal status between groups (all p>0.1). Overall, the vaccine was well tolerated (max local tox: 100% Grade (Gr) 1; max systemic tox: 15% Gr 0, 70% Gr 1, 15% G r2). The LR significantly increased from R1 to R2 (46.8mm+8.6 v 85.4mm+11.8, p<0.05), from R2 to R3 (85.4mm+11.8 v 120.5mm+11.4, p=0.01), and then plateaued R3-R6 (120.6mm+11.4 v 124.4mm+23.9, p=0.97). With five patients completing R6, DTH increased from R0 to R6 (11.8mm+2.1 v 21.3mm+8.1, p=0.14). After a median follow-up of 7 months, there has been 1 recurrence (7.7%) in the vaccine group vs 3 recurrences (25%) in the CG (p=0.32).

## Conclusions

E39 is an immunogenic peptide derived from FRa. Results from the first three dosing cohorts of this phase I trial suggest the E39 vaccine is well tolerated and elicits a strong in vivo immune response against Fra suggesting that an expansion to a phase IIa trial to better evaluate efficacy is warranted.

